# Aging characteristics of colorectal cancer based on gut microbiota

**DOI:** 10.1002/cam4.6414

**Published:** 2023-08-07

**Authors:** Yinhang Wu, Jing Zhuang, Qi Zhang, Xingming Zhao, Gong Chen, Shugao Han, Boyang Hu, Wei Wu, Shuwen Han

**Affiliations:** ^1^ Huzhou Central Hospital Affiliated Central Hospital Huzhou University Huzhou China; ^2^ Key Laboratory of Multiomics Research and Clinical Transformation of Digestive Cancer Huzhou China; ^3^ Fifth Affiliated Clinical Medical College of Zhejiang Chinese Medical University, Huzhou Central Hospital Huzhou China; ^4^ Institute of Science and Technology for Brain‐Inspired Intelligence Fudan University Shanghai China; ^5^ Second Affiliated Hospital of School of Medicine Zhejiang University Hangzhou China

**Keywords:** aging, artificial intelligence, colorectal cancer, gut microbiota, metagenomic sequencing

## Abstract

**Background:**

Aging is one of the factors leading to cancer. Gut microbiota is related to aging and colorectal cancer (CRC).

**Methods:**

A total of 11 metagenomic data sets related to CRC were collected from the R package curated Metagenomic Data. After batch effect correction, healthy individuals and CRC samples were divided into three age groups. Ggplot2 and Microbiota Process packages were used for visual description of species composition and PCA in healthy individuals and CRC samples. LEfSe analysis was performed for species relative abundance data in healthy/CRC groups according to age. Spearman correlation coefficient of age‐differentiated bacteria in healthy individuals and CRC samples was calculated separately. Finally, the age prediction model and CRC risk prediction model were constructed based on the age‐differentiated bacteria.

**Results:**

The structure and composition of the gut microbiota were significantly different among the three groups. For example, the abundance of *Bacteroides vulgatus* in the old group was lower than that in the other two groups, the abundance of *Bacteroides fragilis* increased with aging. In addition, seven species of bacteria whose abundance increases with aging were screened out. Furthermore, the abundance of pathogenic bacteria (*Escherichia_coli, Butyricimonas_virosa*, *Ruminococcus_bicirculans*, *Bacteroides_fragilis* and *Streptococcus_vestibularis*) increased with aging in CRCs. The abundance of probiotics (*Eubacterium_eligens*) decreased with aging in CRCs. The age prediction model for healthy individuals based on the 80 age‐related differential bacteria and model of CRC patients based on the 58 age‐related differential bacteria performed well, with AUC of 0.79 and 0.71, respectively. The AUC of CRC risk prediction model based on 45 disease differential bacteria was 0.83. After removing the intersection between the disease‐differentiated bacteria and the age‐differentiated bacteria from the healthy samples, the AUC of CRC risk prediction model based on remaining 31 bacteria was 0.8. CRC risk prediction models for each of the three age groups showed no significant difference in accuracy (young: AUC=0.82, middle: AUC=0.83, old: AUC=0.85).

**Conclusion:**

Age as a factor affecting microbial composition should be considered in the application of gut microbiota to predict the risk of CRC.

## 1 INTRODUCTION

Aging is the gradual decrease in the body's physiological and psychological adaptation to the environment and the gradual progression toward death. The physiological changes in the aging process of the human body are mainly reflected in the loss of tissue cells and components, the slowing down of metabolic rate, and the decline of body and organ functions.^1^, ^2^ Recent studies have shown that aging is the result of comprehensive factors such as stem cell decline, DNA degeneration, dietary and spiritual factors, and active aging genes. However, there is still no unified theory of aging. Recent mechanisms to explain aging include somatic mutations,^3^ free radical theory,^4^ natural biomolecular crosslinking, and “uncapped” telomere accumulation.^5^ The common hallmarks of aging include genomic instability, telomere attrition, epigenetic alterations, loss of proteostasis, deregulated nutrient sensing, mitochondrial dysfunction, cellular senescence, stem cell exhaustion, and altered intercellular communication.^2^ Studies have shown that aging is a major risk factor for human pathologies, including cardiovascular disease and neurodegenerative disease, malignancies, metabolic diseases, diabetes, and so on.^6^

The global population is getting older. The incidence of cancer increases with aging, indicating that there is a link between aging and cancer. Organism senescence is the senescence of the cells of an organism at the whole, system or organ level, but it is not equal to the senescence of all cells of an organism. Aging individuals are less able to maintain and restore homeostasis in response to stress and injury, thus increasing the risk of malignancy. Inducing senescence of tumor cells is considered an antitumor mechanism.^7^ However, senescent cells can also promote cancer. Increased levels of reactive oxygen species (ROS) due to oxidative stress induced by the accumulation of mitochondrial DNA (mtDNA) mutations that occur in aging have also been found in cancer.^8^ Both cancer and aging can be seen as accumulation of cellular damage. Almost all cancers are caused by somatic mutations, including breast, pancreatic, lung, liver, medulloblastoma, leukemia, and B‐cell lymphoma.^9^ High expression of senescence‐associated secretory phenotypes (SASPs), such as MMP3 (stromelysin)^10^ and VEGF,^11^ is associated with driving the development of cancer. Cancer and aging may share a common origin. Aging is divided into physiological senescence and pathological senescence. Physiological senescence refers to the gradual aging of cells without disease due to a decrease in the body's ability to remove senescent cells. Tumors are manifestations of pathological aging.

Colorectal cancer (CRC) is the third most common malignant tumor in the world, and the occurrence and prevalence of CRC are closely related to age.^12^ More than 90% of CRC tumors are first diagnosed in individuals over 50 years of age.^13^ The incidence of early‐onset CRC (diagnosis at an age of less than 50 years) has significantly increased.^14^ Age is an important factor in recurrence and poor prognosis of CRC.^15^ However, new tumor histopathological evidence suggests that early‐onset CRC is prone to recurrence and poor prognosis.^16^, ^17^ Yue et al. identified 15 aging‐related genes (ITGA7, CD36, PCSK5, FNBP1, etc.) associated with poor prognosis of CRC and 11 aging‐related genes (CCL28, CCNB1, PLK1, MOC1, etc.) associated with good prognosis.^18^ In addition, aging affects CRC tumor immunity. Early‐onset CRC showed low levels of tumor‐infiltrating lymphocytes. Later‐onset CRC showed a higher density of macrophages, M1‐like macrophages, CD14HLA‐DR cells, and CD3CD4FOXP3 cells.^19^ However, the exact relationship between aging and CRC remains unclear. The identification of CRC aging‐related targets may provide new evidence for CRC prognosis and risk assessment.

The abundance of gut microbes is more than 100 times the size of the human genome. Gut microbes encode more than 3.3 million genes.^20^ Colorectal cancer is closely associated with the gut microbiota. The main manifestations of intestinal dysbiosis in CRC are the loss of beneficial bacteria (e.g., *Lactobacillus*, *Granulicatella*, *Proteobacteria*), an increase in pathogens or potentially harmful species (e.g., *Fusobacterium*, *Peptostreptococcus*, *Streptococcus*, *Ruminococcus*, *Bacteroidetes*, Firmicutes), and the loss of overall microbial diversity.^21^ A cohort study found that the intestinal microbiome diversity in those with early‐onset CRC was lower than that of the normal population of the same age, but the diversity was still higher than that of later‐onset CRC. *Flavonifractor plautii* is increased in early‐onset CRC, and *Streptococcus* is increased in later‐onset CRC.^22^ This indicates that age is one of the factors that affects the structure and composition of gut microbiota in CRC samples.

It has been confirmed that the composition of the gut microbiome is different in healthy individuals of different ages.^23^ Intestinal diversity and complexity gradually increase from infancy to adulthood. The gut microbiota is most stable at adulthood. However, after entering senectitude, the diversity of the human gut microbiota decreases, the dominant species and beneficial microorganisms (such as *Firmicutes* and *Actinobacteria*) decrease, and the state of the gut microbiome becomes unstable.^24^ The gut microbiome is a key regulator and indicator of aging. Animal experiments confirmed that the proportion of *Firmicutes*/*Bacteroidetes* in the intestinal tract of elderly individuals was significantly higher than that in the intestinal tracts of younger individuals, indicating that aging is more likely to induce intestinal microecology disorders.^25^

Furthermore, intestinal microecology disorders further damage intestinal permeability, induce systemic inflammation, and more easily lead to adverse health outcomes.^26^ Therefore, we hypothesize that age‐induced intestinal microecological disturbances may influence individual CRC risk. The analysis of age‐related differential gut microbiota in CRC and the age‐related characteristics of CRC can provide a new biological target and accurate means for CRC risk prediction and prognosis assessment.

In the present study, intestinal microbiome metagenomic data were collected from healthy individuals and CRC samples. The structure and composition of the gut microbiota in the three age groups were compared, and the different bacteria from CRC samples from the different age groups were screened. The aim of this study was to confirm the presence of age‐related bacteria in the intestines of CRC patients. It was shown that CRC is an aging‐related disease. The results of this study might provide novel aging‐related biomarkers for CRC risk prediction.

## 2 METHODS

### 2.1 Data acquisition and processing

A total of 11 metagenomic datasets related to CRC were collected from the R package curated Metagenomic Data,^27^ and further screened for samples with detailed age and BMI information. The R package provided MetaPhlAn3 annotated species results. The relative abundance table of species composition at the species level and genus level was obtained for downstream analysis. The samples with <2% species were removed, and the information of the final 1296 samples is summarized in Table S1. After the data from 11 items were used for batch effect correction (MMUPHin package), the species with prevalence <0.01 and maximum relative abundance <0.001 among all samples were filtered out, and the datasets were combined for downstream statistical analysis.

### 2.2 Descriptive analysis

For the combined species, species‐level data were analyzed separately for the health/disease group according to the following procedure. The ggplot2 and MicrobiotaProcess packages were used to visualize species composition and perform principal component analysis (PCA). The top 20 species of bacteria were selected according to the comprehensive ranking of average abundance and prevalence. The composition of CRC samples and samples from healthy individuals were calculated, and a PCA dimensionality reduction graph was produced after the logarithmic relative abundance of species was calculated.

### 2.3 Difference analysis

LEfSe was performed on the three age brackets of the healthy/CRC groups. The microbiomeMarker package was used to test the difference in relative abundance data of species. The different species were screened out with the threshold of effect size LDA Score (log10) > 2. Age‐differentiated bacteria from the healthy group were compared with disease‐differentiated bacteria from all samples. To prevent the difference caused by sample size, all samples were repeatedly downsampled five times (healthy and CRC downsampled 303 times), and the occurrence frequency of markers greater than or equal to three out of five was selected as the stable disease differentiator. Finally, the intersection of the disease differentiator with all samples was selected as the final disease differentiator. The healthy/CRC samples were divided into three age groups, and the differences in bacteria between the healthy and CRC groups were compared for each age group. The method is the same as above.

### 2.4 Correlation analysis

Spearman correlation coefficients were calculated separately for CRC/healthy groups, and FDR multiple test correction was performed by BH. Only the correlations with absolute values of correlation coefficients >0.4 and *p* values <0.05 were retained, and the rest were assigned 0. Plot correlation heatmaps were produced with the corrplot package.

### 2.5 Construction of CRC risk and age prediction model

According to the risk group label of all samples and the age bracket label of the health/disease group, a binary classifier was established using randomForest and caret package three times fivefold nested cross‐validation randomForest algorithm. First, the age prediction model was established (young and middle were combined as the young group). Based on the modeling of age‐differentiated bacteria, nested cross‐validation results were obtained, AUC, F1 score, sensitivity, and specificity were calculated, and a confusion matrix was drawn. The healthy/CRC samples were modeled separately. The top 20 important features of the model were removed, and the intersection was removed. The Wilcoxon difference test and FDR multiple test correction were performed. Finally, the intersection between the disease‐differential bacteria and the age‐differential bacteria in the healthy samples was removed, and the remaining 31 differential bacteria were used to construct the CRC risk prediction model. Moreover, CRC risk prediction models were established for each of the three age groups based on differential disease bacteria in the young group, middle group, and old group.

### 2.6 Statistical analysis

SPSS 22.0 statistical software was used for statistical processing. Continuous variables are expressed as the mean ± standard deviation (mean ± SD). The enumeration data consistent with a normal distribution were compared using the *t* test. Statistical differences of *p* < 0.05 were considered significant.

## 3 RESULTS

### 3.1 Differences in gut microbiota in different ages

We prepared a collection of metagenomic sequences for 691 CRC samples and 605 healthy individuals from 11 publicly available datasets (Table S1). The corrected species and genus data were analyzed in the healthy/CRC group according to the analytical procedure (Figure S1). Pretreatment results showed that species was superior to genus. Histograms of age differences and frequencies of healthy/CRC subgroups were calculated and show that the data are more concentrated in 50–75 years old. The Wilcoxon variance test showed significant age differences among individuals in the healthy/CRC groups. Therefore, the samples were divided into three age groups (≤55, 55–65, and >65 years) to ensure that the number of samples of all ages was similar (Figure S2, Table 1).

**TABLE 1:** Frequency statistics of age groups.

	Young (≤55)	Middle (55, 65)	Old (>65)	
Healthy	193	196	216	605
CRC	153	235	303	691

**FIGURE 1:**
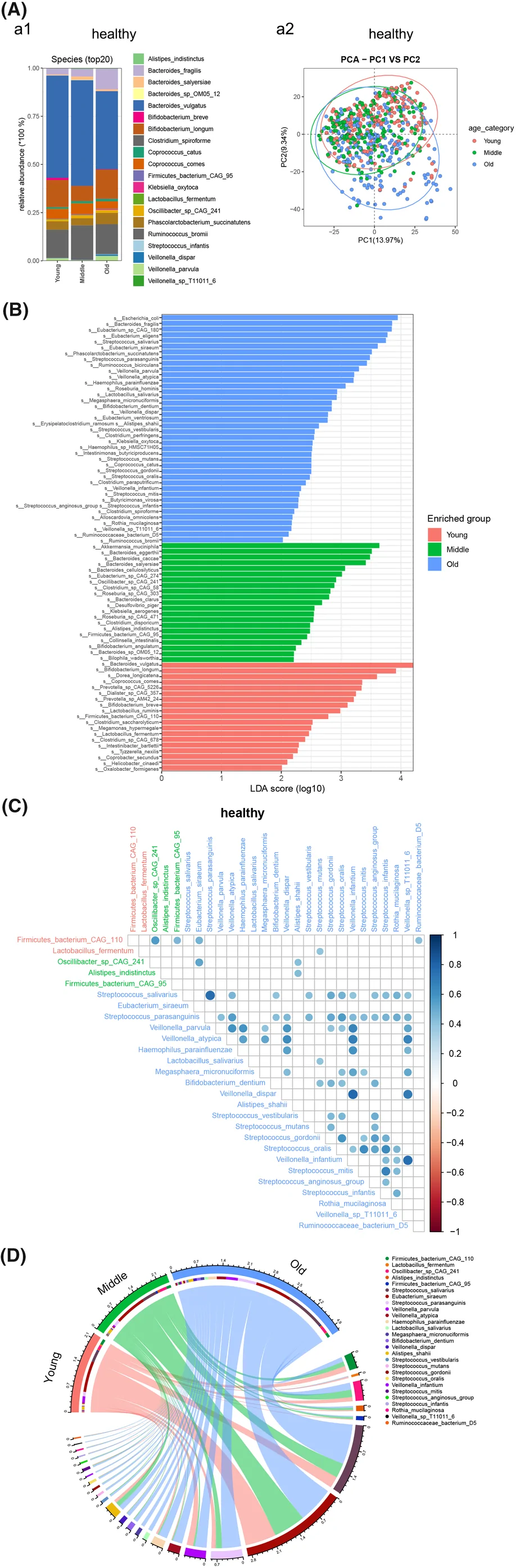
Structure and differences in gut microbiota in the healthy group at different ages. (A‐a1) Average abundance and prevalence ranking of the top 20 bacterial species in the three age groups within the healthy group. (A‐a2) PCA dimension reduction diagram. The horizontal and vertical coordinates are the first and second principal components, respectively (explanatory variance percentage in parentheses). (B) LEfSe hierarchical tree is shown above. Different color nodes indicate the microbial groups that are significantly enriched in the corresponding groups and have a significant influence on the differences between groups. The following is the LDA discriminant histogram. Species with LDA score >2 differences are shown, that is, biomarkers with significant differences. Species with significant differences in abundance in different groups are shown. The length of the bar chart represents the impact of the species with significant differences. The greater the LDA score is, the greater the impact of species abundance on the differential effect. (C) Heatmap of the correlation between different species. The size and color of the corresponding point at each position correspond to its Spearman correlation coefficient. The color of the font corresponds to the direction of enrichment of differentials, which is consistent with the color in the right picture. The young group is red, the middle group is green, and the old group is blue (diagonal elements are not considered). (D) Circos diagram. The average abundance of bacteria with significant age differences among each group of samples.

The structure and composition of the gut microbiota in healthy individuals at young, middle, and old ages were compared. The results showed that the abundance of *Bacteroides vulgatus* in the three age groups was the highest, but the abundance in the old group was lower than that in the other two groups. The relative abundance of *Bacteroides fragilis* increased with aging. The structure and composition of the gut microbiota in the elderly were significantly different from those in the young and middle‐aged groups (Figure 1A).

Further analysis of differences revealed 80 different species of bacteria in the three age groups. The biomarkers in the young group included *Escherichia_coli*, *Bacteroides fragilis*, and *Eubacterium_sp_CAG_180*. The biomarkers in the middle group included *Ruminococcus bromii*, *Akkermansia muciniphila*, and *Bacteroides eggerthii*. The biomarkers in the old group included *Bacteroides vulgatus*, *Bifidobacterium longum*, *Dorea longicatena*, and so on (Figure 1B).

The correlation between different species at three age groups in the healthy population was analyzed. The correlation between *Veillonella_infantium* and *Veillonella_dispar* was the highest (Spearman = 0.786) (Figure 1C). Analyzing correlations between differential species and age, *Streptococcus salivarius*, *Streptococcus parasanguinis*, *Veillonella parvula*, *Veillonella atypica*, *Haemophilus parainfluenzae*, *Lactobacillus salivarius*, *Megasphaera micronuciformis*, *Bifidobacterium dentium*, and *Veillonella dispar* were the most relevant to the older age group. *Lactobacillus salivarius* and *Megasphaera micronuciformis* were only associated with old age. *Eubacterium siraeum* was evenly distributed in the three age groups (Figure 1D).

### 3.2 Age affects the composition of gut microbiota in CRC samples

**FIGURE 2:**
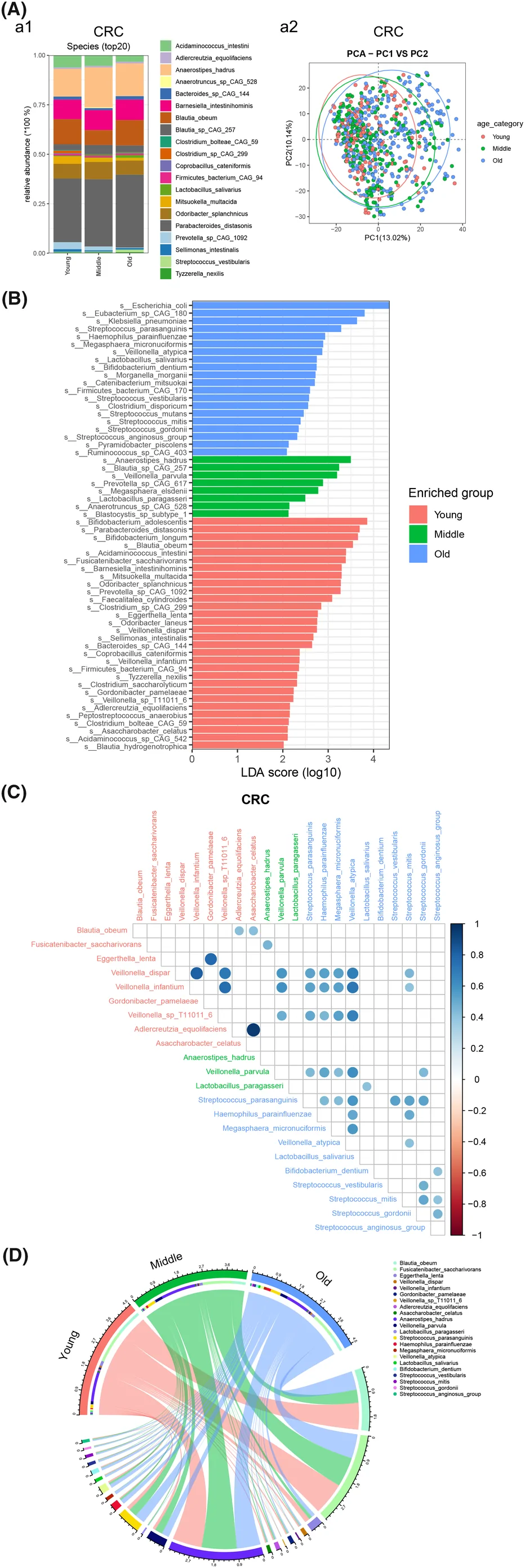
Structure and differences in different gut microbiota of CRC. (A‐a1) Average abundance and prevalence ranking of the top 20 bacterial species in the three age groups of CRCs. (A‐a2) PCA dimension reduction diagram. (B) LEfSe hierarchical tree is shown above. The LDA discriminant histogram is shown below. (C) A heatmap of the correlation between different species. (D) Circos diagram.

The structure and composition of the gut microbiota of the CRC population at young, middle, and old ages were also compared. The results showed that the abundance of *Parabacteroides distasonis* was the highest in the three age groups, and the abundance of *Parabacteroides distasonis* was relatively high in the old group. *Prevotella_sp_CAG_1092, Acidaminococcus_intestini*, and *Mitsuokella_multacida* decreased with aging. The structure and composition of the gut microbiota in CRC samples from the old group were significantly different from those in the young and middle‐aged groups (Figure 2A).

Further differential analysis screened 58 different bacteria among CRC samples of three age groups. The biomarkers in the young group included *Escherichia_coli*, *Eubacterium_sp_CAG_180*, and *Klebsiella pneumoniae*. The biomarkers in the middle group included *Escherichia_coli*, *Eubacterium_sp_CAG_180*, and *Klebsiella pneumoniae*. The biomarkers in the old group included *Bifidobacterium adolescentis*, *Parabacteroides distasonis*, and *Bifidobacterium longum* (Figure 2B).

The correlation between different species in the three age groups of CRC samples was analyzed, and the correlation between *Adlercreutzia equolifaciens* and *Asaccharobacter celatus* was the largest (Spearman = 0.957). *Veillonella_infantium* was also associated with *Veillonella_dispar* (Spearman = 0.815) (Figure 2C). Analyzing the correlation between differential species and the age of CRC samples, we found that *Lactobacillus_paragasseri*, *Streptococcus_parasanguinis*, *Haemophilus_parainfluenzae*, *Megasphaera_micronuciformis*, *Veillonella_atypica*, *Lactobacillus_salivarius*, *Bifidobacterium_dentium*, *Streptococcus_vestibularis*, *Streptococcus_mitis*, *Streptococcus_gordonii*, and *Streptococcus_anginosus_group* were more correlated with CRC samples from the old group than the young and middle‐aged groups. Young CRC samples were not associated with *Lactobacillus_paragasseri*. *Blautia_obeum*, *Fusicatenibacter_saccharivorans*, and *Anaerostipes_hadrus* were evenly distributed in the three age groups and accounted for a significant proportion overall (Figure 2D).

### 3.3 Role of age in CRC intestinal differential bacteria

We intermingled 80 age‐differentiated bacteria from healthy individuals with 58 age‐differentiated bacteria from CRC samples.

**FIGURE 3:**
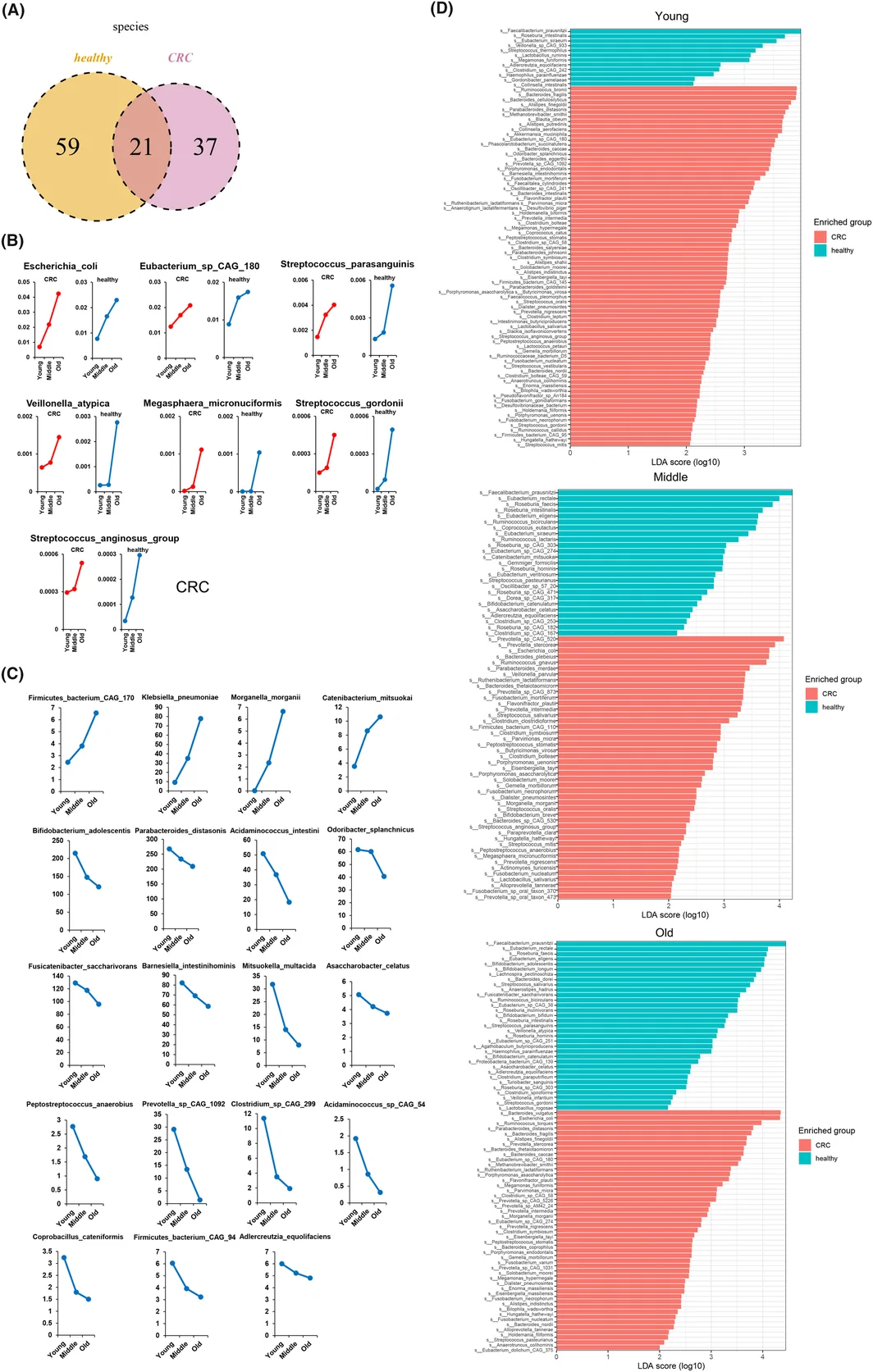
Effect of aging on gut microbiota. (A) Bacterial Venn diagram of age differences between the healthy group and CRC group. (B) Seven age‐differentiated bacteria with increasing abundance with aging in the healthy and CRC groups. (C) Age‐differentiated bacteria with a step‐change in abundance with increasing age that are unique to the CRC group. (D) The differences in gut microbiota in the healthy group and the CRC group (young, middle, and old groups) were analyzed, as shown by the LDA discriminant histogram.

Twenty‐one species of common differential bacteria were identified. Among them, seven species increased in abundance with aging in both populations: *Escherichia_coli*, *Eubacterium_sp_CAG_180*, *Streptococcus parasanguinis*, *Veillonella atypica*, *Megasphaera micronuciformis*, *Streptococcus gordonii*, and *Streptococcus anginosus group* (Figure 3A,B).

In addition, there were 37 age‐related unique bacteria in the CRC samples, excluding the intersecting parts. Among them, 15 types of bacteria decreased with aging in the CRC samples: *Bifidobacterium_adolescentis*, *Parabacteroides_distasonis*, *Acidaminococcus_intestini*, *Fusicatenibacter_saccharivorans*, *Barnesiella_intestinihominis*, *Mitsuokella_multacida*, *Odoribacter_splanchnicus*, *Prevotella_sp_CAG_1092*, *Coprobacillus_cateniformis*, *Firmicutes_bacterium_CAG_94*, *Adlercreutzia_equolifaciens*, *Peptostreptococcus_anaerobius*, *Asaccharobacter_celatus*, and *Acidaminococcus_sp_CAG_542*. Four types of bacteria increased with aging in the CRC samples: *Klebsiella pneumoniae*, *Morganella_morganii*, *Catenibacterium_mitsuokai*, and *Firmicutes_bacterium_CAG_170* (Figure 3C).

Moreover, we divided the healthy and CRC groups into three groups according to age and compared the differential bacteria in each age group (Figure 3D).

Eighty‐eight differentially abundant bacteria were selected from the young group between the healthy and CRC groups. *Faecalibacterium prausnitzii*, *Roseburia intestinalis*, and *Eubacterium siraeum* were enriched in the samples from healthy individuals. *Ruminococcus bromii*, *Bacteroides fragilis*, and *Bacteroides cellulosilyticus* were enriched in CRC samples.

Seventy differentially abundant bacteria were screened out from the middle age group. *Faecalibacterium prausnitzii*, *Eubacterium rectale*, and *Roseburia faecis* were abundant in healthy individuals. *Prevotella_sp_CAG_520*, *Prevotella_stercorea*, and *Escherichia_coli* were enriched in CRC samples.

Eighty differential bacteria were screened out from the old group. *Faecalibacterium prausnitzii*, *Eubacterium rectale*, and *Roseburia faecis* were abundant in healthy individuals. *Bacteroides vulgatus*, *Escherichia_coli*, and *Ruminococcus torques* were enriched in CRC samples.

**FIGURE 4:**
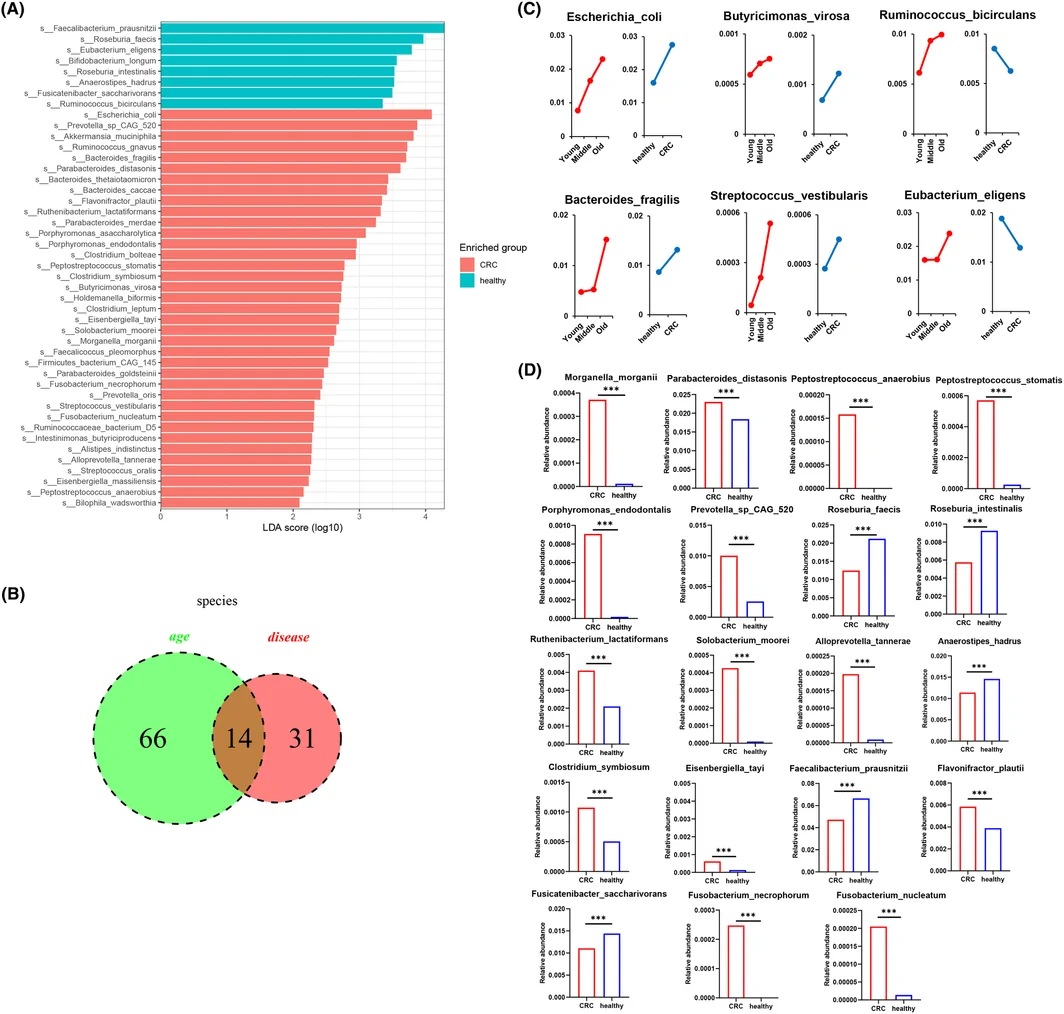
Effect of aging on gut microbiota changes in CRC. (A) LEfSe hierarchical tree. (B) LDA discriminant histogram. (C) Venn diagram of age‐differentiated bacteria and CRC disease‐differentiated bacteria in the healthy group. (D) The abundance of five species increased with aging, and the intestinal abundance of CRC increased. The abundance of one species increased with aging, but the intestinal abundance of CRC decreased. (E) Age‐independent differential bacteria for CRC disease, *** indicating *p* < 0.001.

We also performed an overall disease differential bacterial analysis. Comparing the differential bacteria from healthy individuals and CRC samples, 45 disease‐related differential bacteria were found. Among them, the disease‐related biomarkers included *Escherichia_coli*, *Prevotella_sp_CAG_520*, *Akkermansia muciniphila*, *Ruminococcus gnavus*, and *Bacteroides fragilis*. (Figure 4A).

Finally, we conducted overall disease differential bacterial analysis and found 14 age‐related CRC differential bacteria after the intersection of 80 age‐differential bacteria of healthy individuals with 45 disease‐related differential bacteria (Figure 4B). Five bacteria were identified that increased in intestinal abundance with aging in CRC samples: *Escherichia_coli*, *Butyricimonas violus*, *Ruminococcus bicirculans*, *Bacteroides fragilis*, and *Streptococcus vestibularis*. *Eubacterium_eligens* decreased in the intestinal abundance of CRC samples with aging (Figure 4C). In addition, after adjusting for age, we identified 31 CRC differential bacteria. Among them, 19 bacteria with significant differences (*p* < 0.001) included *Morganella_morganii*, *Peptostreptococcus_anaerobius*, *Peptostreptococcus_stomatis*, and *Fusobacterium_nucleatum*. (Figure 4D).

### 3.4 Establishment of the age prediction model and CRC risk prediction model

**FIGURE 5:**
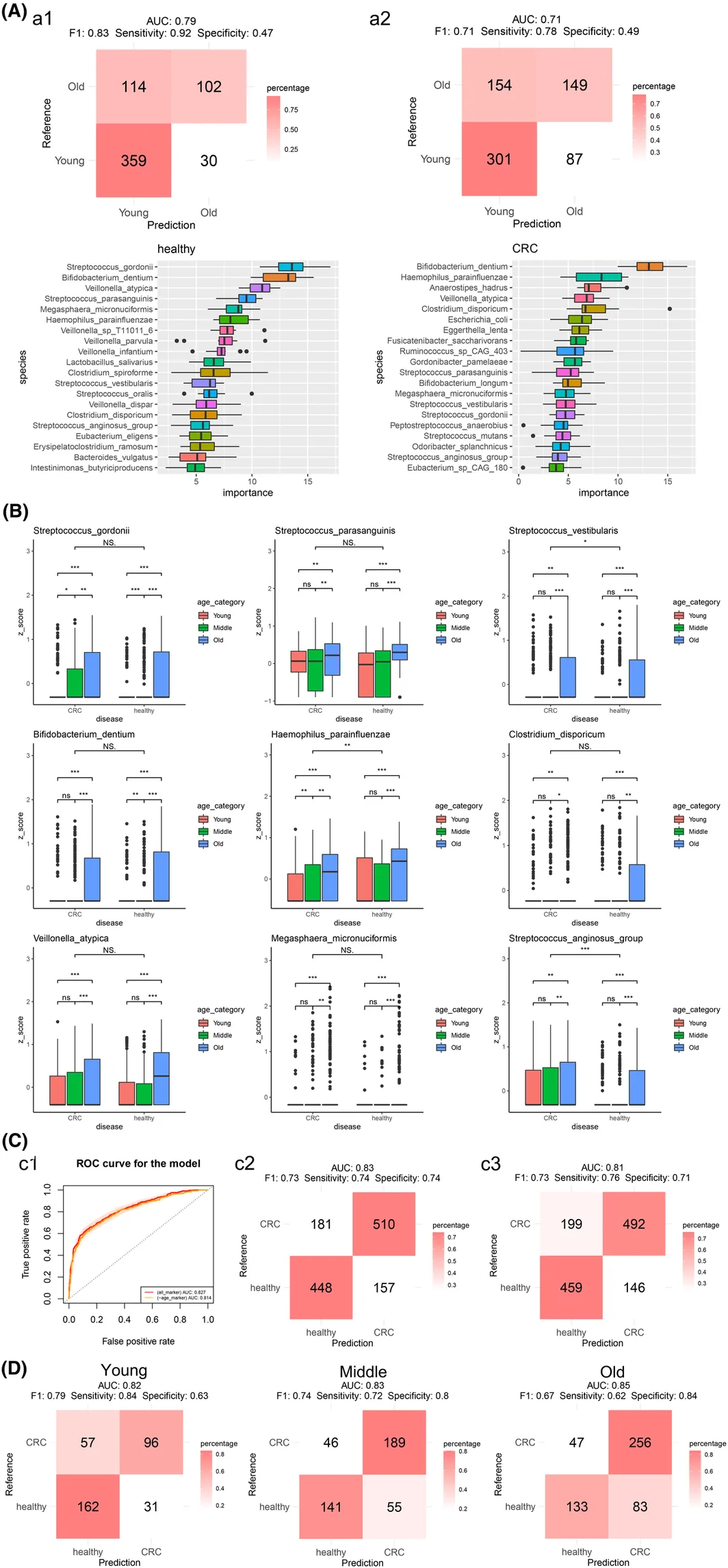
Age prediction model and CRC risk prediction model. (A‐a1) The figure above shows the cross‐validation results of the age prediction model for healthy samples. The following figure is the interpretation diagram of the top 20 feature models, and the horizontal coordinate is relative importance. (B) Box plot and mean difference test of the Z score (logarithmic standardization of relative abundance) for nine overlapping species in each grouping. (C‐c1) CRC risk prediction model based on all samples. The red line “all_marker” represents the use of all 45 disease differential bacteria, and the yellow line “age_marker” represents the use of 31 disease differential bacteria after removing age bacteria. (C‐c2) cross‐validation results of age prediction models for all 45 disease differentials. (C‐c3) cross‐validation of age prediction models for 31 disease‐differential bacteria. (D) cross‐validation results of CRC risk prediction models based on disease‐differentiating bacteria of three age groups.

Finally, the age prediction model of the healthy group was constructed based on the 80 age‐related differential bacteria. Model prediction using the tripartite is not effective, so the dichotomy model was used instead (the age of the young group was ≤65 years old, and the age of the old group was >65 years old). The results showed that the age model had high accuracy (AUC: 0.79, sensitivity: 0.92, specificity: 0.4). In addition, in the age prediction model of healthy individuals, the top three important differential bacteria were *Streptococcus gordonii*, *Bifidobacterium dentium*, and *Veillonella atypica* (Figure 5A‐a1).

A dichotomous model for age prediction of CRC samples was established based on 58 age‐related bacteria (age ≤ 65 years in the young group and >65 years in the old group). The results showed that the age model had high accuracy (AUC: 0.71, sensitivity: 0.78, specificity: 0.49). In addition, in the age prediction model of healthy individuals, the top three important differential bacteria were *Bifidobacterium dentium*, *Haemophilus parainfluenzae*, and *Anaerostipes hadrus* (Figure 5A‐a2).

The intersection of the top 20 important features of the healthy/CRC group age prediction model was removed and tested in groups. *Streptococcus_vestibularis*, *Haemophilus_parainfluenzae*, and *Streptococcus_anginosus_group* were also found to be differential disease bacteria (Figure 5B).

Finally, the CRC risk prediction model results were established based on all samples. An AUC of 0.83 was achieved by using 45 disease‐differential bacteria. After removing the intersection between the disease‐differentiated bacteria and the age‐differentiated bacteria from the healthy samples, the risk prediction model established for the remaining 31 different bacteria showed little difference (in line with expectations), with an AUC of 0.8 (Figure 5C).

To further verify the influence of age on the results of the CRC risk prediction model, we established CRC risk prediction models for each of the three age groups (Figure 5D). The results showed that there was no significant difference in accuracy among the three models (young: AUC = 0.82, middle: AUC = 0.83, old: AUC = 0.85).

## 4 DISCUSSION

The study collected metagenomic data of gut microbiota from 691 CRC samples and 605 healthy individuals and found differences in the structure and composition of gut microbiota between healthy individuals and CRC samples at different ages. In addition, there are differences in bacteria between healthy individuals and CRC samples at different ages. The structure and composition of the gut microbiota in the elderly were significantly different from those in the young and middle‐aged groups. We screened for seven species of bacteria whose abundance increases with aging and are not associated with disease. Furthermore, the abundance of *Escherichia_coli*, *Butyricimonas_virosa*, *Ruminococcus_bicirculans*, *Bacteroides_fragilis*, and *Streptococcus_vestibularis* increased with aging in CRC samples. The abundance of *Eubacterium_eligens* decreased with aging in CRC samples. The age prediction model and CRC risk prediction model based on age‐differentiated bacteria performed well.

Intestinal age, which reflects the changes in the distribution of gut microbiota, is the “third age” of the human body. There are more than 100 species of bacteria, with a total population of approximately 10 trillion in the guts of healthy infants. Normally, the guts of infants are dominated by *Bifidobacterium*, *Lactobacillus*, and *Escherichia_coli*.^28^ As the human body grows, the gut microbiota change. The main factors that determine intestinal age are probiotics such as *Bifidobacterium* and *Lactobacillus*.^29^ The state of intestinal microecology can predict the degree of aging of the body, evaluate the health status of the human body, and measure the incidence of age‐related diseases. Galkin et al. prepared a collection of full intestinal metagenomic sequences for 1165 healthy individuals between 20 and 90 years old and found that the abundance of *Eubacterium hallii* increased with aging and *Bacteroides vulgatus* decreased. At the same time, 39 species of bacteria that could be used to accurately predict biological age were identified.^30^ In the present study, it was found that the abundance of *Bifidobacterium longum* in older ages was different from that in youth or middle age, both in healthy individuals and CRC samples. *Bifidobacterium longum* is a gram‐positive rod‐like bacterium that exists naturally in the human gastrointestinal tract. It is one of the first bacteria to colonize the gut as newborn infants pass through the birth canal.^31^ During adulthood, the levels of *Bifidobacterium longum* decrease considerably but remain relatively stable. It declines progressively in old age.^32^ Chen et al. found that *Bifidobacterium longum* CCFM681 could alleviate colitis by promoting the secretion of conjugated linoleic acid, protecting the intestinal mechanical barrier, modulating the gut microbiota, and inhibiting the TLR4/NF‐κB pathway and associated proinflammatory cytokines.^33^

The structure and composition of the gut microbiota determine intestinal age. Microbiome dysbiosis is a potential hallmark and biomarker of aging.^34^ Age‐related changes in gut microbiota can weaken the gut barrier, releasing toxic products of bacteria, causing inflammation, impairing immune function, and reducing life expectancy.^35^, ^36^ The gut microbiome of older individuals is more complex and unstable than that of younger individuals.^37^ Normally, there is a striking negative association between aging and gut microbe taxonomic richness, diversity, and evenness of the community.^38^ The reason for the longevity may be that the gut microbiota of centenarians tends to be younger in nature. Healthy aging is linked to gut microbiota. The diversity of the gut microbiota increased, and the relative abundance of *Clostridium XIVA*, *Rumenobacteriaceae*, *Akkermansia*, and *Christensenellaceae* was higher among the fecal bacteria of centenarians.^39^ Sato et al. found that centenarians' unique gut microbiome, rich in microbes capable of producing unique secondary bile acids (BAs), protects them from certain bacterial infections, including those caused by multidrug‐resistant bacteria. In addition, *Odoribacteraceae* screened from the intestinal tract of long‐lived elderly people could effectively produce isoalloLCA, a derivative of secondary bile acids, in vitro and in vivo.^40^ Identification of gut microbiota signatures associated with aging provides a promising modulation target for intestinal health.

CRC is a disease highly associated with gut microbes. CRCs are mostly associated with reduced intestinal diversity and intestinal microbial disorders.^41^, ^42^ Aging results in a decrease in beneficial bacteria and an increase in harmful bacteria, such as an increase in *Proteobacteria* and a decrease in *Verrucomicrobia*.^43^ The results of the present study showed that the abundance of *Escherichia_coli*, *Butyricimonas virosa*, *Ruminococcus bicirculans*, *Bacteroides fragilis*, and *Streptococcus vestibularis* increased with aging in CRCs. Among them, *Escherichia_coli* has been shown to affect intestinal barrier integrity, glucose homeostasis, and immune response through the production of aromatic amino acid metabolites. In addition, micromectin produced by *Escherichia_coli* inhibits the growth of other bacteria and secretes the genotoxin colibactin, which causes DNA damage and promotes CRC.^44^ *Escherichia_coli* secretes siderophores, which compete with host cells for iron when iron levels are low during intestinal inflammation, affecting iron uptake by mitochondria. *Escherichia_coli* K‐12 extends the lifespan of the host *C. elegans* by secreting colanic acid to regulate the mitochondrial unfolded protein response (UPRmt) and mitochondrial dynamics.^45^, ^46^ Mitochondria play an important role in the aging process of organisms. Smith et al. found that age‐related mitochondrial DNA mutations cause defects in mitochondrial oxidative phosphorylation (OXPHOS), leading to changes in cellular metabolism and thus accelerating the occurrence of CRC.^47^ Therefore, our results suggested that age should be considered in the study of gut microbiota as a predictor of CRC.

However, there are still some limitations and deficiencies in this study. Considering antibiotics, chemotherapy drugs and other conditions affecting the gut microbiota, strict inclusion and exclusion criteria holds significant importance. The research was based on database analysis, and the data set came from multiple countries. So, strict inclusion and exclusion criteria could not be achieved. In addition, as the data mainly came from public databases, there was a lack of corresponding metabolome data. Therefore, we could not further determine the correlation between metabolic disorders caused by intestinal ecological disorders and the occurrence of CRC through metabolome data. In the future, we will further expand the sample size and carry out multicenter and multi‐omics clinical studies to further confirm the relationship between metabolome and CRC. Furthermore, in view of CRC and age‐related differential bacteria, it is necessary to further explore the specific mechanism by which bacteria cause CRC by influencing aging.

## 5 CONCLUSION

Overall, the age prediction model of healthy individuals and the CRC risk prediction model based on age‐differentiated bacteria have good performance. The present study confirmed that intestinal microbiota are an important characteristic of aging. Age should be considered in the study of gut microbiota as a predictor of CRC. These findings may provide insights into CRC and aging from the perspective of gut microbiota.

## AUTHOR CONTRIBUTIONS

**Yinhang Wu:** Formal analysis (equal). **Jing Zhuang:** Formal analysis (equal). **Qi Zhang:** Writing – original draft (equal). **Xingming Zhao:** Writing – original draft (equal). **Gong Chen:** Investigation (equal). **Shugao Han:** Investigation (equal). **Boyang Hu:** Investigation (equal). **Wei Wu:** Writing – review and editing (equal). **Shuwen Han:** Writing – review and editing (equal).

## FUNDING INFORMATION

This work was supported by the Key Research and Development Project of Zhejiang Province (No. 2022C03026), Zhejiang Medical and Health Technology Project (No. 2022KY1218 and 2023RC274), and Public Welfare Technology Application Research Program of Huzhou (No. 2022GZB04).

## CONFLICT OF INTEREST STATEMENT

The authors declare that no potential conflicts of interest exist.

## CONSENT FOR PUBLICATION

Not applicable.

## ETHICS APPROVAL AND CONSENT TO PARTICIPATE

Not applicable.

## Supporting information


**Figure S1** Flow chart of this study.

**Figure S2** Sample data processing. (A) PCoA diagram (Bray distance) before and after batch effect correction. Different colors indicate different batches, and dot shapes indicate different disease states. Multivariate permutation ANOVA (Vegan package) was conducted, and it was found that although the difference was still significant, the explanatory variance caused by batch decreased, and the center of the 68% confidence ellipse was more concentrated, indicating the effectiveness of batch effect correction. (B) Age distribution chart. (C) Age differences and frequency histogram for disease/health subgroups. (D,E) Age distribution for disease/health subgroups in in different countries.

**Table S1** Basic information for 1296 samples.

## Data Availability

The datasets generated during the current study are not publicly available but obtained from corresponding authors on reasonable request.
