# Supplementary figures and images for: Aging characteristics of colorectal cancer based on gut microbiota
Source: Cancer Med. 2023 Aug 7;12(17):17822–34. doi: 10.1002/cam4.6414 (PMC10524056; doi:10.1002/cam4.6414)

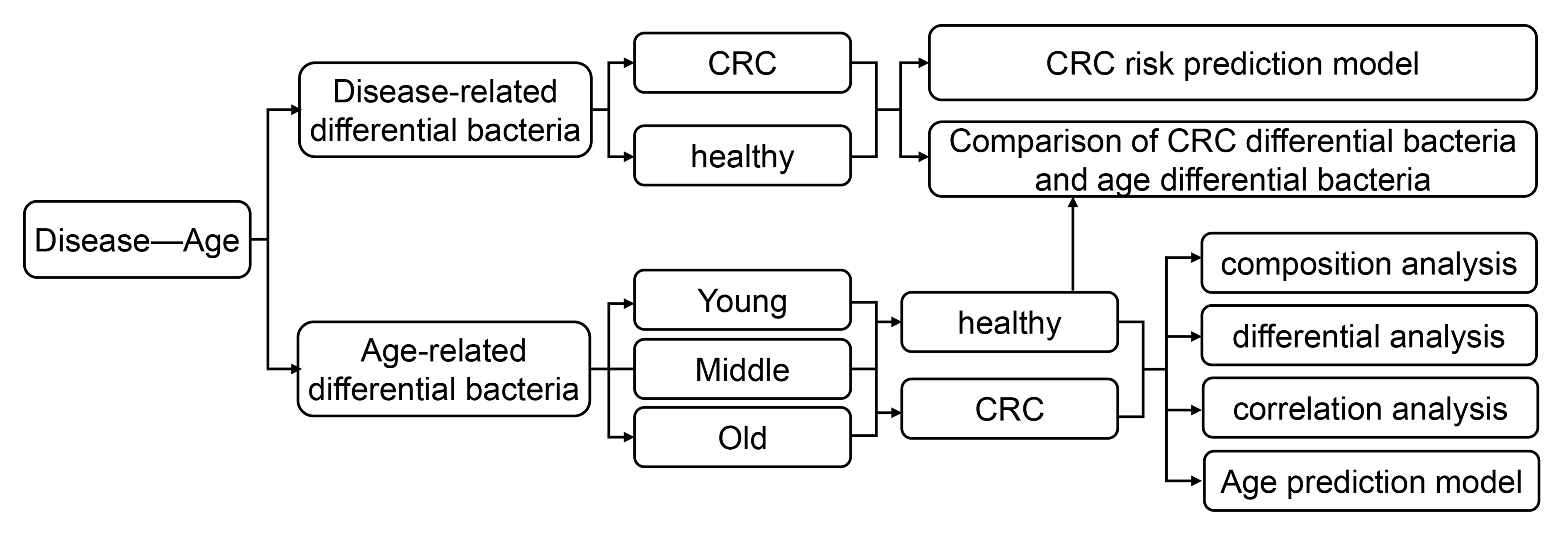

Supplement: Supplementary file 1 — Figure S1 Flow chart of this study. [file CAM4-12-17822-s001.tif]

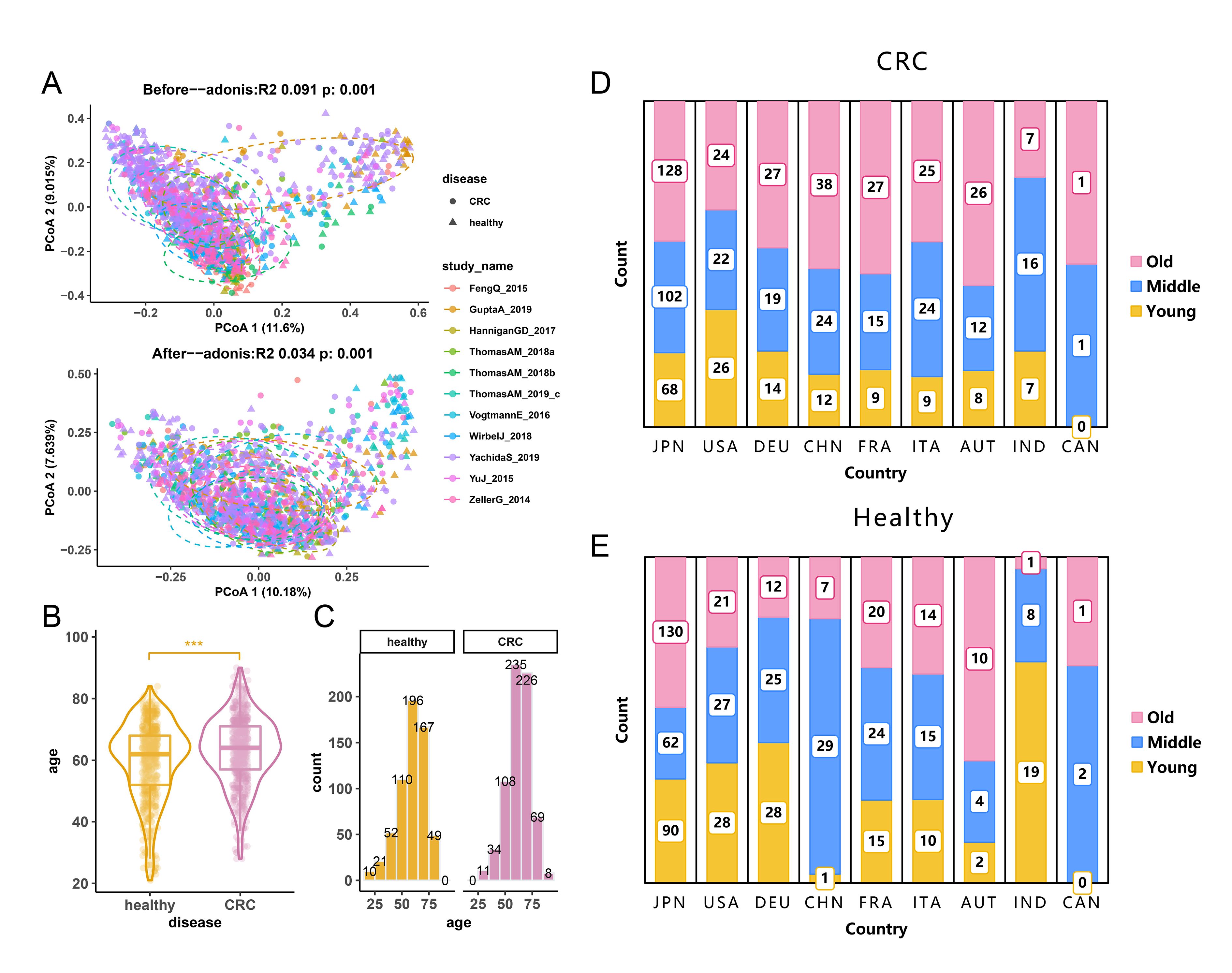

Supplement: Supplementary file 2 — Figure S2 Sample data processing. (A) PCoA diagram (Bray distance) before and after batch effect correction. Different colors indicate different batches, and dot shapes indicate different disease states. Multivariate permutation ANOVA (Vegan package) was conducted, and it was found that although the difference was still significant, the explanatory variance caused by batch decreased, and the center of the 68% confidence ellipse was more concentrated, indicating the effectiveness of batch effect correction. (B) Age distribution chart. (C) Age differences and frequency histogram for disease/health subgroups. (D,E) Age distribution for disease/health subgroups in in different countries. [file CAM4-12-17822-s002.tif]
