# Supplementary material for: Aging characteristics of colorectal cancer based on gut microbiota
Source: Cancer Med. 2023 Aug 7;12(17):17822–34. doi: 10.1002/cam4.6414 (PMC10524056; doi:10.1002/cam4.6414)
Supplement: Supplementary file 3 — Table S1 Basic information for 1296 samples. [file CAM4-12-17822-s003.docx]

**Table S1 Basic information for 1296 samples**

| Study | CRC | Healthy | Country |
| --- | --- | --- | --- |
| Feng Q_2015 | 46 | 16 | AUT |
| Gupta A_2019 | 30 | 28 | IND |
| Hannigan GD_2017 | 25 | 27 | USA, CAN |
| Thomas AM_2018a | 27 | 12 | ITA |
| Thomas AM_2018b | 31 | 27 | ITA |
| Thomas AM_2019c | 40 | 40 | JPN |
| Vogtmann E_2016 | 49 | 52 | USA |
| Wirbel J_2018 | 60 | 65 | DEU |
| Yachida S_2019 | 258 | 242 | JPN |
| Yu J_2015 | 74 | 37 | CHN |
| Zeller G_2014 | 51 | 59 | FRA |
| Total | 691 | 605 |  |
